# First Case Report of Human Plague Caused by Excavation, Skinning, and Eating of a Hibernating Marmot (*Marmota himalayana)*

**DOI:** 10.3389/fpubh.2022.910872

**Published:** 2022-05-26

**Authors:** Jinxiao Xi, Ran Duan, Zhaokai He, Lei Meng, Daqin Xu, Yuhuang Chen, Junrong Liang, Guoming Fu, Li Wang, Hua Chun, Shuai Qin, Dongyue Lv, Hui Mu, Deming Tang, Weiwei Wu, Meng Xiao, Huaiqi Jing, Xin Wang

**Affiliations:** ^1^Gansu Provincial Center for Disease Control and Prevention, Lanzhou, China; ^2^State Key Laboratory of Infectious Disease Prevention and Control, National Institute for Communicable Disease Control and Prevention, Chinese Center for Disease Control and Prevention, Beijing, China; ^3^Shenzhen Nanshan Maternity and Child Health Care Hospital, Shenzhen, China; ^4^Subei Mongolian Autonomous County Center for Disease Control and Prevention, Jiuquan, China; ^5^Jiuquan Municipal Center for Disease Control and Prevention, Jiuquan, China

**Keywords:** hibernation, plague control, *Yersinia pestis*, *Marmota himalayana*, case report

## Abstract

**Introduction:**

The Qinghai-Tibet Plateau is considered the most plague-heavy region in China, and skinning and eating marmots (*Marmota himalayana*) are understood to be the main exposure factors to plague. *Yersinia pestis* is relatively inactive during marmots' hibernation period. However, this case report shows plague infection risk is not reduced but rather increased during the marmot hibernation period if plague exposure is not brought under control.

**Case Presentation:**

The patient was a 45-year-old man who presented with high fever, swelling of axillary lymph nodes, and existing hand wounds on his right side. *Y. pestis* was isolated from his blood and lymphatic fluid. Hence, the patient was diagnosed with a confirmed case of bubonic plague. Later, his condition progressed to septicemic plague. Plague exposure through wounds and delays in appropriate treatment might have contributed to plague progression.

**Conclusion:**

This case report reveals that excavating a hibernating marmot is a significant transmission route of plague. Plague prevention and control measures are priority needs during the marmot hibernation period.

## Introduction

Plague is a highly virulent zoonotic disease mainly transmitted by fleabites and contact with contaminated animal tissues (1). Skinning and eating *Marmota himalayana* is a major transmission route of plague in the *M. himalayana* plague focus, where plague is most active and virulent in China (2). During marmot hibernation, the risk of plague infection seems to be reduced owing to no marmot activity or contact with humans. However, in this case report, we show that there is a high risk of human plague when hibernating marmots are excavated.

## Case Presentation

### Clinical Manifestation, Diagnosis, and Treatment

**Figure 1:**
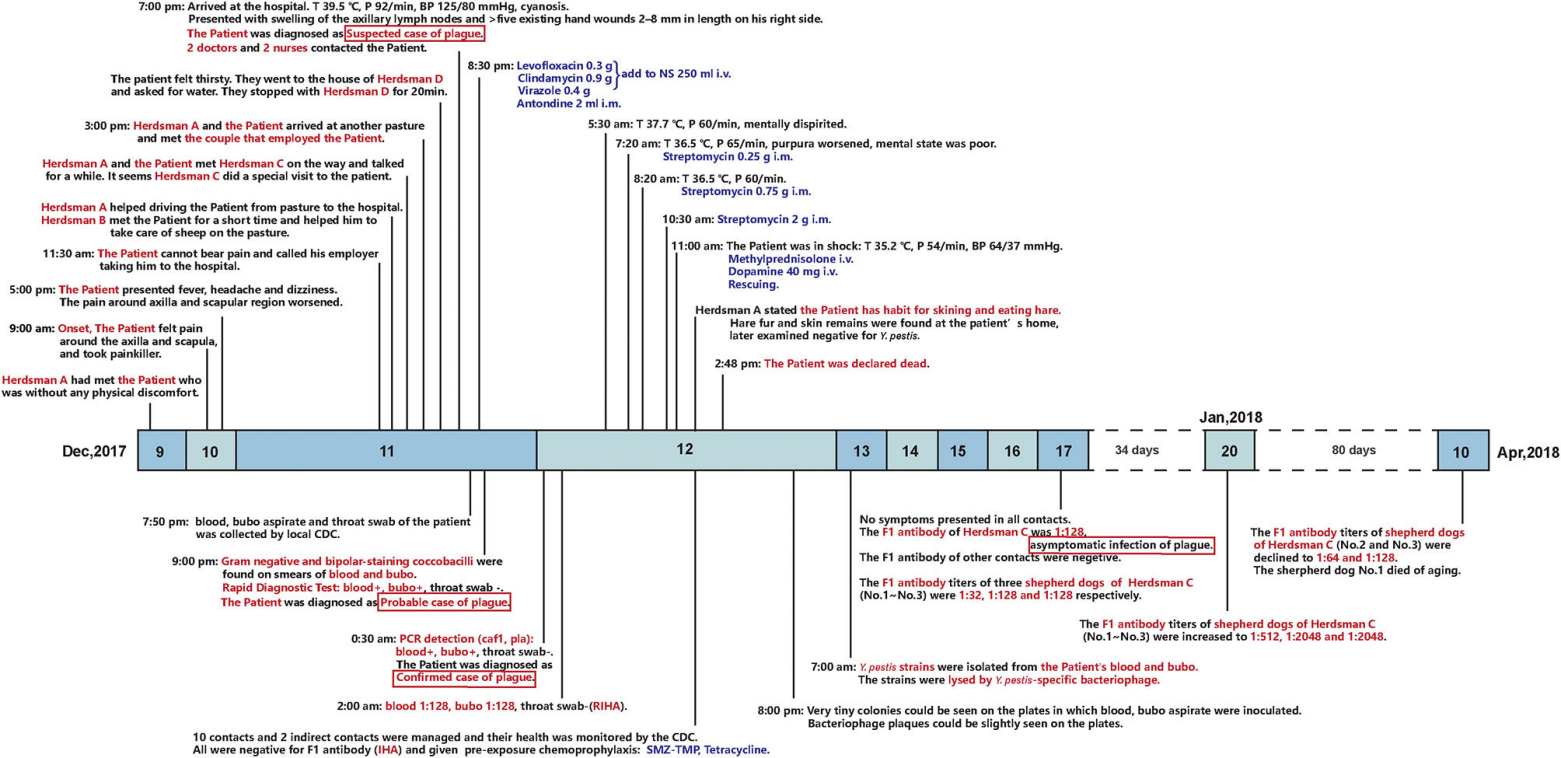
Timeline of the outbreak and follow-up on shepherd dogs. Above the timeline: epidemiological history and clinical presentations of the patient. Below the timeline: laboratory tests of the patient and the contacts, post-exposure prophylaxis of the contacts, and follow-up on the anti-F1 antibody titer of the three shepherd dogs.

The patient was a 45-year-old man who was a non-local and employed as a long-term shepherd herding sheep. On 10 December, his shoulder started to hurt, and he took some analgesic medication. One day later, the pain became unbearable, and he decided to go to a hospital, for which he spent > 7 h along a 170 - km rugged mountain road. In the county hospital, he presented with high fever, slight cyanosis, swelling of the axillary lymph nodes, and > 5 existing hand wounds measuring 2–8 mm in length on his right side, but he had no respiratory symptoms. He was suspected of having plague. A total of.3 g levofloxacin was administrated intravenously. On 12 December, the diagnosis of bubonic plague was confirmed after laboratory examination, including *Y. pestis* culturing, PCR, and the Fraction 1 (F1) antigen of s detection. At 0.720 h on 12 December, he showed symptoms of septic shock, such as mental confusion and worsened purpura, and progressed to septicemic plague. Because the patient was allergic to streptomycin, streptomycin was administrated at.25, 75, and 2 g intramuscularly (Figure 1). At 1100 h on 12 December, the patient went into shock, and clinical rescue failed.

### Laboratory Detection

Gram staining of blood and lymphatic fluid samples showed bacilli with bipolar staining and blunt ends. The strains isolated from both samples were lysed by a *Y. pestis*-specific bacteriophage. The genes *caf1* and *pla* (3) were positive in both samples but negative in the throat swab. Consistently, the titer of the F1 antigen in both samples was 1:128 but negative in the throat swab (4). According to different region (DFR) analysis, the isolate was Genomovar 8, the main type of the local *Y. pestis* strain (5).

### Epidemiological Sourcing

A total of 10 close contacts were identified: a couple that employed the patient, four herdsmen, and four medical staff (Figure 1). All the contacts received pre-exposure chemoprophylaxis, isolation management, and health monitoring according to the plague control guidelines of China (6, 7). The anti-F1 antibody of all the contacts was negative (titer, ≤ 1:4) (8, 9) on 12 December. On 17 December, the titer of herdsman C was 1:128, as he showed no other clinical manifestations, he was diagnosed with an asymptomatic plague infection. The titer of his three dogs were 1:32, 1:128, and 1:128, respectively.

According to epidemiology investigation and laboratory evidence, it was indicated that herdsman C was exposed to plague at the same time as the patient. According to herdsman A's statement, the patient often skinned and ate hare, which was later proven by the remains of hare fur and skin found at the patient's home, but all samples were negative for *Y. pestis*. Before 17 December, herdsman C denied coming into contact with a marmot but later cooperated with the investigation of his dogs for fear of legal punishment. After his and his dogs' antibody titers turned positive, he admitted that the patient and himself had been exposed to a hibernating marmot. They lived on adjacent pastures and had excavated, skinned, and consumed a hibernating marmot. The marmot's viscera had been fed to herdsman C's dogs at his home.

The patient was most likely infected through contact with contaminated fluid or tissue *via* his wounds, but the possibility of flea-borne transmission cannot be ruled out. Gastrointestinal infection is less likely, as the cooking was thorough. Lastly, the patient showed no signs of pneumonic plague. Thorough inspections were conducted around the two men's houses, but no marmot leftover was found. Several *Ochotona curzoniae* (commonly known as pika) but not plague hosts were caught alive around the patient's house, but all tested negative for *Y. pestis*.

## Discussion and Conclusion

Plague infection risk is greatly increased by excavating hibernating marmots. *Y. pestis* can lie dormant in an otherwise highly susceptible host during hibernation, and it is, therefore, hard to tell whether a hibernating marmot is a plague carrier. The *M. himalayana* plague focus of the Qinghai–Tibet plateau has been constantly active and the leading source of human plague in China for decades (10, 11). Most *Y. pestis* strains were isolated from marmots found dead in the environment (2). However, marmot health cannot be assessed during hibernation. The temperature of the burrow is consistently <10°C (12), which largely inhibits the growth of *Y. pestis*. However, in winter-sleeping animals in the torpor state, the level of innate immunity decreases, and animals are more susceptible to plague infection (13). During hibernation, marmots are in a state of heterothermia, wherein the physiological state of torpor (body temperature 5–10°C) is replaced by a state of euthermia (37°C) up to 15–20 times (14–16). When marmot body temperature restored to 28°C and then 37°C, growth and virulence expression of *Y. pestis* (17) are accumulated in marmots. In addition, marmot families hibernate together; thus, more than one marmot can be found at a time in a single burrow (18). These factors increase the risk of plague infection when hibernating marmots are excavated.

In this report, different exposure levels and antibiotic administration led to distinct outcomes. First, a shorter incubation period and a probably higher *Y. pestis* load were found in the patient than in herdsman C because of multiple existing wounds on the patient's hand. In contrast, no obvious wound was found on Herdsman C's hands, who also did less manual work as the owner of another pasture. Second, the timing and use of antibiotics were different. The patient was first administrated levofloxacin.3 g, and then after septic shock, streptomycin was prescribed, which may have further exacerbated the sepsis. Although streptomycin was administrated by gradually increasing the dose to avoid allergy, the contribution of allergy to death cannot be excluded. In contrast, the local herdsman C may have taken effective antibiotics after his special visit to the patient on 11 December. A plague first-aid kit is available at each herdsman's house in China, including sulfamethoxazole-trimethoprim (SMZ-TMP) and oxytetracycline. On 12 December, herdsman C also took SMZ-TMP and tetracycline under CDC management. It was presumed that he recovered before symptoms occurred because of the antibiotics.

This case report reveals that excavating a hibernating marmot is a significant transmission route of plague. The report also dissects related risk factors and provides public health interventions. First, stronger supervision against the illegal acquisition of marmots is needed, especially during the hibernation period. With education on self-protection and ban on marmot hunting, trafficking, and sale, the infection rate in poachers and herdsmen has dropped sharply. However, employed shepherds have become the victims. These people likely receive less education on self-protection and engage in marmot consumption. In this case, the local herdsman C knew that digging a hibernating marmot was forbidden, so he denied the exposure at first. It was only when his and his dogs' antibody titers became positive that he admitted to touching and consuming a marmot. Second, there is an urgent need to improve basic medical services in the *M. himalayana* plague focus. Medical facilities are very limited in this vast focus and mostly very basic. The patient spent too much time getting to the hospital for treatment. Insufficient chemotherapy and unsuccessful use of streptomycin were the key features of this non-pneumonic case. A sharp contrast is no death from pneumonic plague in the United States in 2014 (19). A pneumonic case transferred to Beijing from *Meriones unguiculatus* plague focus was cured there, where effective treatment was adopted (20).

This case report poses new challenges to plague prevention and control of exposure to hibernating marmots. Plague infection risk is not reduced but rather increased during marmot hibernation if plague exposure is not brought under control. In addition, the ability of primary doctors is in need of urgent improvement for plague diagnosis and treatment.

## Data Availability Statement

The datasets presented in this study can be found in online repositories. The names of the repository/repositories and accession number(s) can be found in the article/supplementary material.

## Ethics Statement

The studies involving human participants were reviewed and approved by Ethics Committee of the National Institute for Communicable Disease Control and Prevention of the Chinese Center for Disease Control and Prevention. The patients/participants provided their written informed consent to participate in this study. The animal study was reviewed and approved by Ethics Committee of the National Institute for Communicable Disease Control and Prevention of the Chinese Center for Disease Control and Prevention. Written informed consent was obtained from the owners for the participation of their animals in this study.

## Author Contributions

JX, DX, GF, LW, HC, and HJ were engaged in the outbreak investigation. JX, LM, JL, and XW interpreted the patient data. RD, SQ, DL, HM, DT, WW, MX, HJ, and XW followed up with the shepherd dogs. JX, RD, ZH, and XW were major contributors in writing the manuscript. HJ and XW received the funding. All authors contributed to the article and approved the submitted version.

## Funding

This study was supported by the National Science and Technology Major Project (2018ZX10713-003-002 and 2018ZX10713-001-002). The funding sources for this study had no role in the design of the study and collection, analysis, and interpretation of data, and in writing the manuscript.

## Conflict of Interest

The authors declare that the research was conducted in the absence of any commercial or financial relationships that could be construed as a potential conflict of interest.

## Publisher's Note

All claims expressed in this article are solely those of the authors and do not necessarily represent those of their affiliated organizations, or those of the publisher, the editors and the reviewers. Any product that may be evaluated in this article, or claim that may be made by its manufacturer, is not guaranteed or endorsed by the publisher.
